# CYP27A1 expression is associated with risk of late lethal estrogen receptor-positive breast cancer in postmenopausal patients

**DOI:** 10.1186/s13058-020-01347-x

**Published:** 2020-11-11

**Authors:** Siker Kimbung, Maria Inasu, Tor Stålhammar, Björn Nodin, Karin Elebro, Helga Tryggvadottir, Maria Ygland Rödström, Karin Jirström, Karolin Isaksson, Helena Jernström, Signe Borgquist

**Affiliations:** 1 Department of Clinical Sciences Lund, Division of Oncology, Lund University, Barngatan 4, SE-221 85 Lund, Sweden; 2 Department of Clinical Sciences Lund, Division of Oncology and Therapeutic Pathology, Lund University, Lund, Sweden; 3 Department of Reconstructive Plastic Surgery, Skåne University Hospital, Malmö, Sweden; 4 Department of Clinical Sciences Lund, Division of Surgery, Lund University, Lund, Sweden; 5 Department of Surgery, Central Hospital, Kristianstad, Sweden; 6 Department of Oncology, Aarhus University Hospital, Aarhus University, Aarhus, Denmark

**Keywords:** Cholesterol, 27-hydroxycholesterol, CYP27A1, Breast cancer, Prognosis

## Abstract

**Background:**

27-Hydroxycholesterol (27HC) stimulates estrogen receptor-positive (ER+) breast cancer (BC) progression. Inhibiting the sterol 27-hydroxylase (CYP27A1) abrogates these growth-promoting effects of 27HC in mice. However, the significance of CYP27A1 expression on BC biology and prognosis is unclear.

**Methods:**

Intratumoral CYP27A1 expression in invasive BC was measured by immunohistochemistry in two Swedish population-based cohorts (*n* = 645 and *n* = 813, respectively). Cox proportional hazards models were used to evaluate the association between CYP27A1 expression and prognosis.

**Results:**

CYP27A1 was highly expressed in less than 1/3 of the tumors. High CYP27A1 expression was more frequent among high-grade tumors lacking hormone receptor expression and with larger tumor sizes. Over a median of 12.2 years follow-up in cohort 1, high CYP27A1 expression was associated with impaired survival, specifically after 5 years from diagnosis among all patients [overall survival (OS), HR_adjusted_ = 1.93, 95%CI = 1.26–2.97, *P* = 0.003; breast cancer-specific survival (BCSS), HR_adjusted_ = 2.33, 95%CI = 1.28–4.23, *P* = 0.006] and among patients ≥ 55 years presenting with ER+ tumors [OS, HR_adjusted_ = 1.99, 95%CI = 1.24–3.21, *P* = 0.004; BCSS, HR_adjusted_ = 2.78, 95%CI = 1.41–5.51, *P* = 0.003].

Among all patients in cohort 2 (median follow-up of 7.0 years), CYP27A1 expression was significantly associated with shorter OS and RFS in univariable analyses across the full follow-up period. However after adjusting for tumor characteristics and treatments, the association with survival after 5 years from diagnosis was non-significant among all patients [OS, HR_adjusted_ = 1.08, 95%CI = 0.05–2.35, *P* = 0.83 and RFS, HR_adjusted_ = 1.22, 95%CI = 0.68–2.18, *P* = 0.50] as well as among patients ≥ 55 years presenting with ER+ tumors [OS, HR_adjusted_ = 0.46 95% CI = 0.11–1.98, *P* = 0.30 and RFS, HR_adjusted_ = 0.97 95% CI = 0.44–2.10, *P* = 0.93].

**Conclusion:**

CYP27A1 demonstrated great potentials as a biomarker of aggressive tumor biology and late lethal disease in postmenopausal patients with ER+ BC. Future studies should investigate if the benefits of prolonged endocrine therapy and cholesterol-lowering medication in BC are modified by CYP27A1 expression.

## Background

Over 75% of primary breast cancers (BC) express the estrogen receptor (ER) alpha (α) and depend on the ER signaling for sustained growth and survival [1, 2]. Consequently, several targeted therapeutics interfering with ER signaling have been developed and initially are very successful in controlling the ER+ disease. However, a significant number of tumors become resistant to treatment and the patients present with disease recurrences both locally and at distant sites [2]. Metastasis is a principal cause of BC-related death [3]. Importantly, BC is still the leading cause of cancer-related deaths among women worldwide [3]. Understanding the underlying factors promoting disease onset and progression and developing more precise biomarkers and effective therapeutic strategies for early prognostic decision-making and to overcome therapy resistance, remains a pertinent research priority.

Obesity and overweight have been shown to increase the risk of postmenopausal ER+ BC onset and progression by about 50% [4–9]. High cholesterol, which is often associated with overweight/obesity, has also emerged as a risk factor for postmenopausal BC onset and progression [9–11]. Preclinical studies have demonstrated that high cholesterol favors cancer progression in mice [12–14], and molecular profiling studies have shown that cholesterol metabolism is often reprogrammed in many cancer types [15–19]. We and others have reported that the concomitant use of cholesterol-lowering medications during adjuvant therapy for BC is associated with improved survival outcomes, especially among postmenopausal patients with ER+ disease [20–23]. Thus, a distinct role for cholesterol in promoting BC progression has emerged, strongly in favor for the prescription of therapeutics that interfere with cholesterol metabolism in the management of ER+ BC. However, the incomplete understanding of the biochemical mechanisms directly responsible for the pathogenicity of cholesterol in cancer has hindered the progress of this promising therapeutic strategy in the fight against BC.

Nelson and colleagues [12] have validated the previous in vitro data showing that the cholesterol metabolite 27-hydroxycholesterol (27HC) is a potent ERα ligand [24, 25] and have extended these findings to clearly demonstrate that 27HC is capable of promoting ER+ BC progression in mice. Importantly, the ability of 27HC to stimulate the transcriptional activity of ERα leading to BC progression was highly dependent on the concentration of 17β-estradiol in the surroundings; 27HC mainly activated ERα in the absence of estrogen or under hypo-estrogenic conditions while it principally displayed antagonistic effects on ERα under normal to high estrogenic conditions [24–26]. This estrogen-dependent property of 27HC on ERα activity qualifies it as the only characterized endogenously occurring selective estrogen receptor modulator (SERM) with a great potential to differentially impact ER+ BC progression and outcome depending on the patient menopausal status but this remains to be comprehensively investigated in clinical BC.

27HC is synthesized from cholesterol by the sterol 27-hydroxylase (CYP27A1). Nelson et al. have further demonstrated that the tumor growth-enhancing effect of a high cholesterol diet in mice is attributable to 27HC and not directly to cholesterol since the pro-tumorigenic effects of 27HC and a high-cholesterol diet in the mice were abrogated by genetic and pharmacological inhibition of CYP27A1 [12, 27]. These novel results have increased the attention in targeting cholesterol metabolism in BC and have brought CYP27A1 into the repertoire of druggable targets for treating ER+ BC. Translating these interesting preclinical findings into effective treatments is still limited by, among other things, the absence of sensitive assays to measure 27HC levels in very small amounts of material such as the very scarce clinical tumor samples, specific targeted drugs and biomarkers for personalizing treatment. It is still unclear how 27HC levels in the tumor-microenvironment impact the course of BC progression and survival in patients. To uncover relevant associations between tumor pathological features, menopausal status, and BC prognosis in relation to intratumoral CYP27A1 expression, we performed immunohistochemistry to quantify CYP27A1 expression in two separate Swedish population-based prospective cohorts of patients with invasive primary BC.

## Patients and methods

### Study populations

#### Cohort 1: Malmö Diet and Cancer Study (MDCS)

Initially, CYP27A1 expression was quantified in invasive breast tumors from women diagnosed with primary BC during follow-up in the prospective population-based cohort study: Malmö Diet and Cancer Study (MDCS). The MDCS prospectively enrolled 28,098 participants from the Malmö municipality, of which 17,035 were women, to study associations between diet and cancer. Recruitment of study participants was initiated in March 1991 and closed in September 1996. Detailed information regarding MDCS study design, inclusion and exclusion criteria, and data collection has been reported previously [28, 29]. From March 1991 through December 2010, 1016 women with incident BCs were identified through record linkage. Patient and tumor characteristics at the time of BC diagnosis were obtained from medical records and vital status are ascertained by record linkage with the Swedish Cancer Registry and the Southern Swedish Regional Tumor Registry [30, 31].

#### Cohort 2: Breast Cancer-Blood (BC-Blood) study

To validate and extend our findings, CYP27A1 expression was also assessed in an independent cohort of female patients diagnosed with primary BC at the Skåne University Hospital in Lund, BC-blood study. This prospective population-based cohort recruited patients between October 2002 and August 2019. All participants filled in an extensive questionnaire including reproductive and lifestyle factors and medication use, including statins. Body and weight measurements were collected by a research nurse at study inclusion prior to surgery and at each scheduled postoperative visit to complement standard clinical examination procedures. Other patient and tumor characteristics at time of BC diagnosis were obtained from medical records [32–34]. From study onset in October 2002 through June 2012, 1116 patients were included and a detailed description of the tumor and patient characteristics for this subset has been previously reported [35–38].

#### Patient selection for the current analyses

**Fig. 1:**
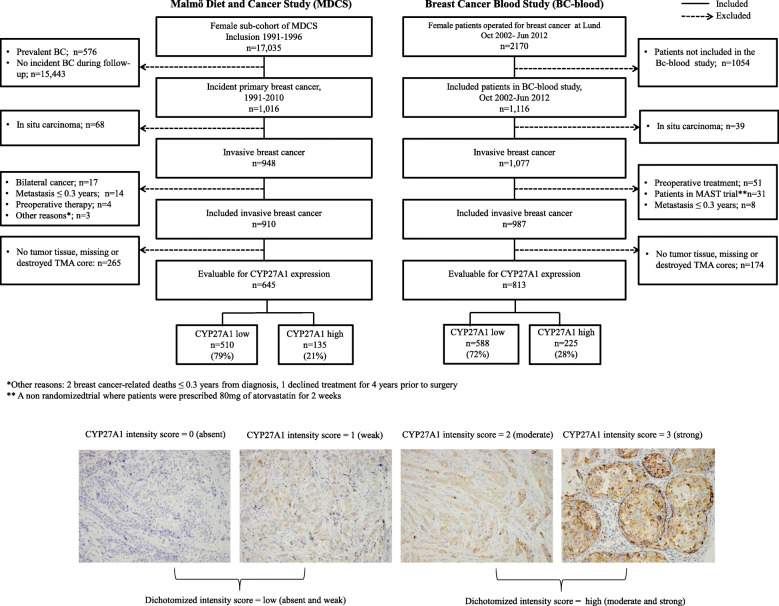
Consort flow diagram of the patient sub-populations included in this study

Herein, we focus on invasive primary BCs only; patients with ductal carcinoma in situ only, who received preoperative treatment, or who had a distant metastatic event or died ≤ 0.3 years after diagnosis were excluded. Consequently, 910 and 987 patients with invasive primary BC remained in the MDCS and BC-blood cohorts, respectively, and were eligible for inclusion in our analyses. Archived formalin-fixed paraffin-embedded (FFPE) blocks were collected wherever possible, and tissue microarrays (TMAs) were constructed for each cohort (Fig. 1).

### Immunohistochemistry (IHC)

TMAs including two 1 mm cores from representative regions of each tumor were constructed for each cohort. IHC staining and evaluation of CYP27A1 expression was performed as previously described [12, 39]. Briefly, 4 μm sections were cut from FFPE TMA blocks, de-paraffinized, treated with antigen retrieval buffer (citrate, pH 6) for 20 min, and then reacted with the anti-CYP27A1 antibody for 2 h (ab126785, Abcam; 1:300 dilution). CYP27A1 expression was evaluated only in tumor cells, and a patient was considered to have a CYP27A1-positive tumor when a granular cytoplasmic reactivity was observed in at least 20% of tumor cells in all evaluable cores for that patient. Staining in stroma cells was not considered. A semi-quantitative intensity score, 0 (absent), 1 (weak), 2 (moderate), or 3 (strong), was adopted for annotating the staining intensities to further differentiate the results. Two investigators blinded to other pathological and survival data performed the IHC evaluation, with guidance from a pathologist. In cases of disagreement (MDCS 6% and BC-blood 9%), a consensus score was established. For statistical analysis, the intensity scores were summarized into a dichotomized variable; low (absent and weak) and high (moderate and strong). Finally, due to no archival FFPE blocks, missing cores, non-intact or poor-quality cores, lack of tumor tissue in the core, and or low cellularity, CYP27A1 expression was successfully evaluated in 71% (645/910) and 82% (813/987) of the eligible invasive primary tumors in MDCS and BC-blood, respectively (Fig. 1). The REMARK guidelines for biomarker assessment and reporting were respected in the analyses presented in this report [40].

### Endpoints

The primary endpoint explored in this study was overall survival (OS), defined as time from BC diagnosis until death due to any cause. Other endpoints explored were breast cancer-specific survival (BCSS; defined as the time from BC diagnosis to death from a BC-related cause only) and recurrence-free survival (RFS; defined as time from BC diagnosis until earliest occurrence of invasive locoregional recurrence, distant metastasis, or death from any cause).

### Statistical analyses

The distribution of patient and tumor characteristics in relation to CYP27A1 expression are presented as percentages (categorical variables) or median and interquartile range (continuous variables). For MDCS power calculations including 600 patients, of which 20% had a high intratumoral expression of CYP27A1 and with a median survival time of 10 years for patients with low intratumoral CYP27A1 expression, it was possible to detect true HRs of ≤ 0.71 or ≥ 1.36 with a probability (power) of 0.8. Similarly, for BC-blood study power calculations including 800 patients, of which 25% had a high intratumoral expression of CYP27A1 and with a median survival time of 7 years for patients with low intratumoral CYP27A1 expression, it was possible to detect true HRs of ≤ 0.75 or ≥ 1.38 with a probability (power) of 0.8. The type I error probability associated with this test of the null hypothesis that the experimental and control survival curves are equal is 0.05. The power calculations were performed with the PS Power and Sample Size Calculation Program, version 3.1.2 [41].

Unexpectedly, shorter storage duration of FFPE block, calculated as the time between surgery and IHC staining for CYP27A1, was statistically significantly associated with high CYP27A1 expression intensity in both cohorts (Table 1). Hence, all associations between CYP27A1 expression and tumor-pathological and patient characteristics reported in this study have been adjusted for the FFPE block storage duration (continuous variable) using logistic regression and Cox regression models, respectively. The impact of CYP27A1 expression is presented as odds ratios (OR) or hazard ratios (HR) and 95% confidence intervals (CI) where applicable. Multivariable models were adjusted for age at diagnosis (age < 55 years vs age ≥ 55 years), tumor size > 20 mm (yes/no), axillary lymph node involvement (yes/no), histological grade (III vs. I and II), ER status (≤ 10% vs > 10%), adjuvant breast cancer therapy [radiotherapy (yes/no), chemotherapy (yes/no), and endocrine therapy (yes/no)], and storage time of FFPE block (continuous). No statistically significant evidence for non-proportional hazards was found in univariate Cox regression models for any of the endpoints assessed (*P* > 0.05 for all proportional hazard tests). Of note, we used the cut-off of 55 years for dichotomizing patient age to serve as a proxy for postmenopausal status in this study since menopausal status was not precisely assessed at the time of primary BC diagnosis. All statistical tests were two-sided, and *P* < 0.05 was considered to be statistically significant. All analyses presented in this study are exploratory and are not adjusted for multiple testing.

**Table 1:** Baseline tumor and patient characteristics in relation to CYP27A1 expression

	MDCS			BC-blood		
	CYP27A1 low	CYP27A1 high	OR *(95% CI)	CYP27A1 low	CYP27A1 high	OR* (95% CI)
**Characteristic**	*N* (%)	*N* (%)		*N* (%)	*N* (%)	
	510 (79.1)	135 (20.9)		588 (72.3)	225 (27.7)	
**Age at BC diagnosis**	64.9 (60.1–71.1)^a^	66.2 (60.4–73.5)^a^	1.02 (0.99–1.05)	60.7 (52.4–67.8)^a^	61.3 (52.3–69.2)^a^	1.00 (0.98–1.01)
Age at diagnosis < 55 yrs	53 (10)	10 (7.4)	ref	179 (30.4)	74 (32.9)	ref
Age at diagnosis ≥ 55 yrs	457 (90)	125 (92.6)	0.94 (0.44–2.00)	409 (69.6)	151 (67.1)	0.84 (0.60–1.18)
**Estrogen receptor status**						
Positive	428 (89.7)	94 (78.3)	ref	545 (92.7)	166 (74.1)	ref
Negative	49 (10.3)	26 (12.6)	**2.52 (1.50–4.20)**	43 (7.3)	58 (25.9)	**4.75 (3.05–7.41)**
**Progesterone receptor status**						
Positive	243 (56.4)	52 (47.3)	ref	445 (75.7)	135 (60.3)	ref
Negative	188 (43.6)	58 (52.7)	**1.81 (1.16–2.84)**	143 (24.3)	89 (39.7)	**2.17 (1.55–3.03)**
**Tumor size**						
≤ 20 mm	353 (69.6)	92 (68.1)	ref	446 (75.9)	150 (66.7)	ref
> 20 mm	154 (30.4)	43 (31.9)	1.10 (0.73–1.70)	142 (24.1)	75 (33.3)	**1.62 (1.15–2.27)**
**Nodal status**						
Negative	304 (63.3)	90 (68.7)	ref	351 (59.8)	144 (64.3)	ref
Positive	176 (36.7)	41 (31.3)	0.81 (0.54–1.23)	236 (40.2)	80 (35.7)	0.84 (0.61–1.16)
**Nottingham histological grade**						
I and II	369 (73.9)	82 (62.6)	ref	466 (79.3)	134 (59.6)	ref
III	130 (26.1)	49 (37.4)	**1.70 (1.13–2.60)**	122 (20.7)	91 (40.4)	**2.37 (1.69–3.32)**
**FFPE block storage time, yrs**	15.0 (12.0–19.0)^a^	13.0 (10.0–17.0)^a^	**0.93 (0.89–0.97)**	10.0 (7.0–12.0)^a^	8.0 (6.0–11.0)^a^	**0.87 (0.82–0.92)**
**Radiotherapy**						
No	160 (34.3)	55 (44.4)	ref	214 (36.4)	78 (34.7)	ref
Yes	307 (65.7)	69 (55.6)	**0.65 (0.43–0.97)**	374 (63.6)	147 (65.3)	1.06 (0.76–1.47)
**Endocrine therapy**						
No	204 (40.6)	50 (37.9)	ref	158 (26.9)	84 (37.3)	ref
Yes	298 (59.4)	82 (62.1)	0.93 (0.61–1.40)	429 (73.1)	140 (62.5)	**0.62 (0.45–0.87)**
**Chemotherapy**						
No	388 (82.9)	97 (78.9)	ref	457 (77.7)	149 (66.2)	ref
Yes	80 (17.1)	26 (21.1)	1.23 (0.74–2.03)	131 (22.3)	76 (33.8)	**1.51 (1.07–2.14)**

^a^Median (IQR), *OR* odds ratio, *ref* reference category, *FFPE* formalin-fixed paraffin-embedded, *yrs* years. Bold numbers indicate statistically significant comparisons

*Odds ratios are only adjusted for storage time of FFPE blocks

## Results

### Similarities and differences in patient and tumor characteristics at baseline between the two cohorts

The distributions of patients, tumor characteristics, and adjuvant treatment were compared between the overall populations and the subsets of patients with evaluable CYP27A1 expression within each cohort and were found to be well-balanced (Supplementary Table 1) for the majority of factors assessed. However, important differences in patient and tumor characteristics and treatment history were noted between the cohorts. The median age at diagnosis was higher in MDCS (65.1 years; IQR = 60.2–71.9 years) compared to BC-blood (60.9 years; IQR = 52.4–68.1 years). An overwhelming majority (90.2%) of the patients in MDCS were 55 years and older (presumably postmenopausal) compared to 68.8% in BC-blood. Although the proportion of patients with ER+ tumors was similar between the two cohorts (87%), endocrine therapy and chemotherapy were more generously used in BC-blood (70.2% and 25.5%, respectively) compared to those in MDCS (59.9% and 17.9%, respectively). Further, the proportion of tumors lacking progesterone receptor (PgR) expression was higher in MDCS (45.5%) compared to that in BC-blood (28.6%).

### CYP27A1 expression in relation to tumor and patient characteristics

The distribution of tumor characteristics in relation to CYP27A1 expression is presented in Table 1. High CYP27A1 expression was observed in about 21% and 28% of tumors in MDCS and BC-blood, respectively (Table 1). In both cohorts, high CYP27A1 tumors were more likely to lack hormone receptor (ER and PgR) expression and to have Nottingham histological grade III (*P* < 0.05 for all comparisons). In BC-blood, high CYP27A1 expression was also significantly associated with larger tumor size (*P* = 0.02) and patients with high CYP27A1 tumors were more likely to have received chemotherapy (OR = 1.51, 95%CI = 1.07–2.14) and less likely to have received endocrine treatment (OR = 0.62, 95%CI = 0.45–0.87). In MDCS, radiotherapy treatment was less frequent among patients with high CYP27A1 tumors (OR = 0.65, 95%CI = 0.43–0.97).

In the BC-blood study, where preoperative weight, height, and waist and hip circumferences were measured, there were no statistically significant associations between high CYP27A1 expression and body mass index ≥ 25 kg/m^2^ or waist-to-hip ratio > 0.85 (both *P* > 0.05, Supplementary Table 2). Similarly, there was no association between self-reported preoperative statin use and CYP27A1 expression in the BC-blood study (*P* = 0.45). Similarly, in MDCS, there was no association between CYP27A1 expression and history of statin use according to data retrieved from the Swedish Prescribed Drug Register and Swedish Death Registry (*P* = 0.24).

### CYP27A1 expression and long-term prognosis in the MDCS cohort

The significance of CYP27A1 expression in relation to survival was first explored among all eligible patients, followed by pre-specified sub-analyses stratified by age at diagnosis (age < 55 years vs age ≥ 55 years) and ER expression (≤ 10% vs > 10%). OS and BCSS were the primary and secondary endpoints, respectively. At the end of follow-up (December 31, 2016), 221 deaths had been registered among the 645 patients eligible for inclusion in survival analyses and approximately half of the deaths (*n* = 112) had BC as an underlying cause. The median follow-up for all patients was 10.8 years (IQR; 7.4–15.2 years) and 12.2 years (IQR; 9.1–16.6 years) for patients who were alive and still at risk at data cut-point.

**Fig. 2:**
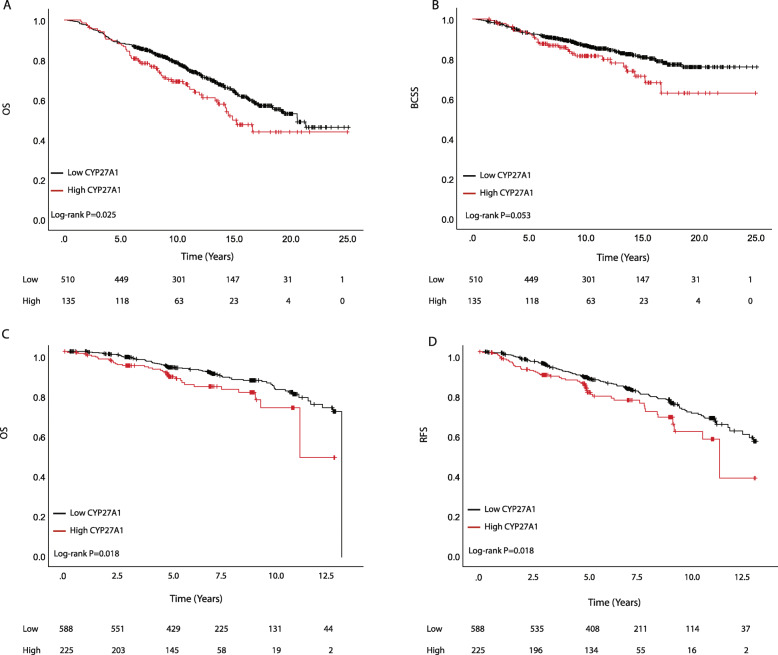
Kaplan-Meier plots showing associations between CYP27A1 expression and survival. **a** Overall survival (OS) and **b** breast cancer-specific survival (BCSS) respectively, for all eligible patients in MDCS. **c** OS and **d** recurrence-free survival (RFS) respectively, for all eligible patients in BC-blood

High CYP27A1 expression was associated with poorer OS and BCSS, specifically impacting the period after 5 years from primary diagnosis (Fig. 2a, b). In Cox regression analyses adjusted for FFPE block storage duration only and including all eligible patients, both OS and BCSS were statistically significantly worse for patients with high CYP27A1 tumors [OS; HR = 1.38, 95%CI = 1.01–1.89, *P* = 0.04 and BCSS; HR = 1.60, 95%CI = 1.01–2.39, *P* = 0.04; respectively]. Similar trends were obtained in sub-analyses among all patients with ER+ tumors [OS; HR = 1.50, 95%CI = 1.04–2.20, *P* = 0.03 and BCSS; HR = 1.53, 95%CI = 0.89–2.63, *P* = 0.12; respectively]. In fully adjusted multivariable Cox models covering the entire follow-up period, CYP27A1 emerged as an independent high-risk factor, increasing the average risk for all-cause mortality by 40% for all patients and by 60% for all ER+ patients and also for women older than 55 years of age with ER+ disease. Likewise, the average risk of dying specifically due to a BC-related cause remained elevated by about 40% for all patients and between 60 and 70% for ER+ patients with high CYP27A1 compared to low CYP27A1 tumors, although the evidence was weaker statistically (Supplementary Table 3).

Postmenopausal patients presenting with ER+ primary breast tumors are recommended 5 years of endocrine therapy as the backbone of their adjuvant cancer treatment regimen, and currently extended adjuvant therapy is suggestively discussed with the patient [42]. The separation of the survival curves at 5 years seen in the previous analyses is therefore remarkable because it coincides with the important milestone for patients with ER+ BCs when adjuvant cancer treatment often ends. We therefore performed a 5-year landmark multivariable Cox analysis to specifically compare the risks of an event before and after 5 years from BC diagnosis by CYP27A1 expression. Interestingly, no statistically significant differences in the risks for an OS or BCSS event were found for the first 5 years as expected, but the risks for an OS event increased above 75%‚ while the risks for BCSS events more than doubled for patients with high CYP27A1 tumors (Table 2). Importantly, CYP27A1 was the only independent factor for both OS and BCSS in the fully adjusted multivariable models (model 2) including patients with ER+ disease and patients aged ≥ 55 years presenting with ER+ tumors only.

**Table 2:** Adjusted hazard ratios (95%CI) for overall survival and breast cancer-specific survival by tumor CYP27A1 expression in MDCS cohort

	Model 0^a^			Model 1^b^			Model 2^c^		
	Cases	Events	CYP27A1 high vs low HR (95%CI)	Cases	Events	CYP27A1 high vs low HR (95%CI)	Cases	Events	CYP27A1 high vs low HR (95%CI)
**Follow-up period ≤ 5 years**									
**Overall survival**									
All	645	76	0.97 (0.56–1.69)	569	69	0.86 (0.47–1.57)	514	63	0.68 (0.35–1.31)
			*P* = 0.92			*P* = 0.63			*P* = 0.25
ER+	522	52	1.05 (0.53–1.03)	498	51	1.09 (0.54–2.19)	448	47	0.91 (0.43–1.93)
			*P* = 0.87			*P* = 0.81			*P* = 0.81
ER+ and age ≥ 55 yrs	472	51	1.10 (0.55–2.21)	450	50	1.11 (0.55–2.23)	400	46	0.97 (0.46–2.05)
			*P* = 0.78			*P* = 0.76			*P* = 0.93
**Breast cancer-specific survival**									
All	645	46	0.92 (0.44–1.93)	569	41	0.67 (0.30–1.51)	514	38	0.54 (0.22–1.31)
			*P* = 0.83			*P* = 0.34			*P* = 0.17
ER+	522	26	0.61 (0.18–2.03)	498	26	0.63 (0.19–2.10)	448	25	0.57 (0.16–1.95)
			*P* = 0.42			*P* = 0.45			*P* = 0.37
ER+ and age ≥ 55 yrs	472	25	0.64 (0.19–2.16)	450	25	0.64 (0.19–2.14)	400	24	0.63 (0.18–2.15)
			*P* = 0.47			*P* = 0.47			*P* = 0.46
**Follow-up period > 5 years**									
**Overall survival**									
All	567	145	1.66 (1.14–2.43)	498	129	1.89 (1.25–2.85)	449	123	1.93 (1.26–2.97)
			*P* = 0.009			*P* = 0.003			*P* = 0.003
ER+	468	122	1.76 (1.14–2.71)	445	117	1.79 (1.16–2.77)	399	112	1.78 (1.13–2.80)
			*P* = 0.01			*P* = 0.009			*P* = 0.01
ER+ and age ≥ 55 yrs	419	112	1.85 (1.18–2.91)	398	107	1.91 (1.22–3.03)	352	102	1.99 (1.24–3.21)
			*P* = 0.007			*P* = 0.005			*P* = 0.004
**Breast cancer-specific survival**									
All	567	66	2.18 (1.28–3.73)	498	60	2.25 (1.26–4.01)	449	58	2.33 (1.28–4.23)
			*P* = 0.004			*P* = 0.006			*P* = 0.006
ER+	468	54	2.22 (1.19–4.12)	445	54	2.18 (1.18–4.03)	399	52	2.26 (1.20–4.27)
			*P* = 0.01			*P* = 0.01			*P* = 0.01
ER+ and age ≥ 55 yrs	419	46	2.56 (1.33–4.91)	398	46	2.57 (1.34–4.94)	352	44	2.78 (1.41–5.51)
			*P* = 0.005			*P* = 0.005			*P* = 0.003

*HR* hazard ratio, *CI* confidence interval, *ER+* estrogen receptor positive

^a^Model 0: adjusted for FFPE block storage duration

^b^Model 1: adjusted for age at diagnosis, tumor size, lymph node involvement, ER expression, tumor histological grade and FFPE block storage duration

^c^Model 2: Model 1 + adjusted for local (radiotherapy) and systemic (endocrine and chemotherapy) treatments

In analogy, CYP27A1 expression did not impact ER-negative BC prognosis, but the analysis was under-powered due to few cases (*N* = 75, data not shown). Similarly, due to too few cases (*N* = 65), a meaningful assessment of the prognostic impact of CYP27A1 could not be performed in the age group < 55 years.

### CYP27A1 expression and long-term prognosis in the BC-blood cohort

To validate and extend our findings from MDCS, CYP27A1 expression in relation to OS and disease progression (RFS) were examined in the BC-blood cohort. At data cut-point (June 30, 2016), 108 OS events and 175 RFS events had been recorded among the 813 patients with valid CYP27A1 expression data. The median follow-up for all patients was 7.0 years (IQR; 4.2–9.1 years) and 7.0 years (IQR; 5.0–9.1 years) for patients who were alive and still at risk at data cut-point.

In the general population across the full follow-up period, both RFS and OS were significantly worse for patients with high CYP27A1 compared to low CYP27A1 tumors [RFS: HR = 1.58, 95%CI = 1.13–2.21, *P* = 0.008 and OS: HR = 1.73, 95% CI = 1.14–2.64, *P* = 0.011] in Cox regression analyses adjusted for FFPE storage duration. Comparable results were obtained in Kaplan-Meier analyses shown in Fig. 2c, d. An analogous non-significant trend towards poorer survival for patients with high CYP27A1 tumors was observed among patients with ER+ tumors [RFS: HR = 1.34, 95%CI = 0.89–2.01, *P* = 0.16; and OS: HR = 1.47, 95%CI = 0.86–2.49, P = 0.16, respectively]. Also, CYP27A1 expression was not significantly associated with prognosis among women aged ≥ 55 years presenting with ER+ tumors [RFS: HR = 1.10, 95%CI = 0.67–1.80, *P* = 0.70; OS: HR = 1.28, 95%CI = 0.70–2.36, *P* = 0.42, respectively]. Notably, among younger patients < 55 years (presumably pre- and perimenopausal) presenting with ER+ tumors, overexpression of CYP27A1 was associated with over twofold increased risk of recurrence [RFS, HR = 2.19, 95%CI = 1.05–4.59, *P* = 0.04] and death [OS, HR = 3.27, 95%CI = 0.99–10.79, *P* = 0.05]. CYP27A1 expression did not impact ER-negative breast cancer prognosis (data not shown). Adjusted HRs for OS and RFS for the full follow-up are presented in Supplementary Table 4. Though showing similar trends, CYP27A1 was not an independent prognostic factor in any of the multivariable models and patient subgroups. Furthermore, the risk estimates remained essentially the same after further adjustments for body mass index, waist-to-hip ratio, or preoperative statin use (data not shown).

The association between CYP27A1 expression and survival was also investigated in BC-blood using 5-year landmark multivariable Cox analyses (Table 3). No statistically significant difference in OS was observed for any of the Cox models or time periods. RFS event rates were, however, found to be more than three-folds significantly higher in patients < 55 years presenting with ER+ tumors during the first 5 years after BC diagnosis only. RFS differences were statistically non-significant for all other analyses.

**Table 3:** Adjusted hazard ratios (95%CI) for overall survival and recurrence-free survival by tumor CYP27A1 expression in BC-blood cohort

	Model 0^a^			Model 1^b^			Model 2^c^		
	Cases	Events	CYP27A1 high vs low HR (95%CI)	Cases	Events	CYP27A1 high vs low HR (95%CI)	Cases	Events	CYP27A1 high vs low HR (95%CI)^b^
**Follow-up period ≤ 5 years**									
**Overall survival**									
All	808	61	1.90 (1.12–3.23)	805	59	1.40 (0.77–2.42)	804	59	1.31 (0.74–2.32)
			*P* = 0.02			*P* = 0.28			*P* = 0.35
ER+	697	41	1.84 (0.93–3.62)	695	40	1.90 (0.96–3.77)	694	40	1.93 (0.97–3.85)
			*P* = 0.07			*P* = 0.06			*P* = 0.06
ER+ and age ≥ 55 yrs	486	37	1.75 (0.84–3.63)	484	36	1.80 (0.86–3.74)	483	36	1.79 (0.85–3.75)
			*P* = 0.13			*P* = 0.12			*P* = 0.12
ER+ and age < 55 yrs	208	4	4.21 (0.56–31.78)	208	4	3.65 (0.31–42.97)	208	4	2.02 (0.13–30.70)
			*P* = 0.16			*P* = 0.30			*P* = 0.61
**Recurrence-free survival**									
All	808	99	1.76 (1.15–2.71)	805	97	1.32 (0.84–2.07)	804	97	1.32 (0.83–2.08)
			*P* = 0.01			*P* = 0.23			*P* = 0.24
ER+	704	72	1.50 (0.87–2.60)	702	71	1.46 (0.85–2.51)	701	71	1.51 (0.88–2.60)
			*P* = 0.14			*P* = 0.17			*P* = 0.13
ER+ and age ≥ 55 yrs	490	54	1.12 (0.58–2.17)	488	53	1.15 (0.59–2.24)	487	53	1.16 (0.60–2.26)
			*P* = 0.73			*P* = 0.67			*P* = 0.66
ER+ and age < 55 yrs	214	18	3.25 (1.20–8.76)	214	18	3.18 (1.08–9.37)	214	18	3.59 (1.15–11.20)
			*P* = 0.02			*P* = 0.03			*P* = 0.03
**Follow-up period > 5 years**									
**Overall survival**									
All	570	47	1.50 (0.72–2.93)	569	46	1.14 (0.54–2.43)	568	46	1.08 (0.50–2.35)
			*P* = 0.29			*P* = 0.73			*P* = 0.83
ER+	509	40	0.96 (0.39–2.35)	508	39	0.77 (0.29–2.01)	507	39	0.79 (0.30–2.08)
			*P* = 0.93			*P* = 0.59			*P* = 0.64
ER+ and age ≥ 55 yrs	348	31	1.46 (0.43–4.92)	347	30	0.42 (0.10–1.82)	346	30	0.46 (0.11–1.98)
			*P* = 0.54			*P* = 0.25			*P* = 0.30
ER+ and age < 55 yrs	124	9	0.38 (0.08–1.70)	124	9	1.60 (0.35–7.46)	124	9	1.69 (0.33–8.73)
			*P* = 0.41			*P* = 0.55			*P* = 0.53
**Recurrence-free survival**									
All	527	74	1.3 (0.77–2.31)	522	75	1.19 (0.67–2.12)	521	75	1.22 (0.68–2.18)
			*P* = 0.29			*P* = 0.54			*P* = 0.50
ER+	474	67	0.83 (0.45–1.54)	470	68	1.01 (0.53–1.90)	469	68	1.05 (0.56–2.00)
			*P* = 0.56			*P* = 0.99			*P* = 0.87
ER+ and age ≥ 55 yrs	328	51	0.91 (0.44–1.91)	324	51	0.91 (0.42–1.98)	323	51	0.97 (0.44–2.10)
			*P* = 0.81			*P* = 0.82			*P* = 0.93
ER+ and age < 55 yrs	123	16	0.66 (0.21–2.08)	124	17	1.10 (0.35–3.51)	124	17	1.24 (0.36–4.20)
			*P* = 0.48			*P* = 0.87			*P* = 0.73

*HR* hazard ratio, *CI* confidence interval, *ER+* estrogen receptor positive

^a^Model 0: adjusted for FFPE block storage duration

^b^Model 1: adjusted for age at diagnosis, tumor size, lymph node involvement, ER expression, tumor histological grade and FFPE block storage duration

^c^Model 2: Model 1 + adjusted for local (radiotherapy) and systemic (endocrine and chemotherapy) treatments

## Discussion

We evaluated the significance of CYP27A1 expression in invasive BC among patients from two independent population-based cohort studies in Southern Sweden. Intratumoral CYP27A1 expression was associated with unfavorable tumor characteristics and significantly impaired survival with differential effects observed between the time periods 0–5 years and greater than 5 years after diagnosis based on menopausal status. After adjusting for potential confounders, the risk for lethal disease among patients with high CYP27A1 tumors remained very significantly elevated in the MDCS cohort for all endpoints and subgroups, especially among older patients ≥ 55 years presenting with ER+ BC, distinctively impairing survival only after 5 years from primary tumor diagnosis.

In the recent past, a distinct role for cholesterol in promoting ER+ BC progression has emerged in preclinical studies, unraveling the promising connections between 27HC, CYP27A1, and ER+ breast cancer progression. In this study, tumor-specific CYP27A1 expression was used as a surrogate for intratumoral 27HC levels to investigate the impact on tumor biology and outcome in clinical BC. The significant associations between high intratumoral levels of CYP27A1 with aggressive breast tumor biological features seen in this study are consistent with previous reports [12, 39] and align with the preclinical evidence supporting a pathogenic role of the 27HC/CYP27A1 metabolic pathway in BC [12, 27, 43].

For the first time and in population-based cohorts, we report a positive association between high CYP27A1 levels and increased risk for late lethal disease, especially among presumably postmenopausal patients presenting with ER+ BC. Prominently, the effect of CYP27A1 expression on prognosis was comparable in magnitude between the OS and BCSS. If high CYP27A1 expression translates into high 27HC, this association with poor survival is thus consistent with the expected agonist activity of 27HC under the hypo-estrogenic physiological state prevalent in postmenopausal patients. Under these conditions, 27HC can still promote ER+ BC progression even under the pressure of endocrine therapies like aromatase inhibitor treatments since it can freely access the unaffected estrogen receptor driving the transcription of tumor growth-promoting genes. Adjusting for adjuvant treatment did not impact the association between CYP27A1 and survival suggesting that 27HC may indeed compromise survival and treatment efficacy in postmenopausal patients. We could, however, not evaluate if CYP27A1 modified the effects of specific types of endocrine therapies in MDCS because the data were not available but considering that over 90% of the patients were above 55 years old at time of diagnosis, it is likely that aromatase inhibitors may have been the treatment of choice for the patients diagnosed from 2009 and onwards when aromatase inhibitors became standard of care for postmenopausal patients. It has been reported that CYP27A1 activity can be inhibited in vitro by anastrozole but not by exemestane and letrozole [44] suggesting another level of complexity to the prognostic impact of CYP27A1 in postmenopausal BC that needs to be investigated.

The observation that the adverse prognostic impact of CYP27A1 expression was specifically prominent 5 years after diagnosis is remarkable since this corresponds to the time period when adjuvant endocrine therapy usually ends. Although the evidence currently indicates that extending AI-based treatment beyond 5 years does not reduce late recurrence risk in an unselected population of patients with ER+ BC [45–47], subset analyses indicated that patients with ER+/PgR-positive, node-positive disease might benefit from extended AI-based treatment.

Metastasis is the principal cause of BC-related death. CYP27A1 may also serve as a marker for selecting patients for extended adjuvant treatment trials in BC. Recently, it was confirmed in the large randomized BIG 1-98 trial that the use of cholesterol-lowering drugs significantly improved survival in postmenopausal women regardless of whether the treatment was initiated before or during adjuvant endocrine therapy [20], but whether delaying the initiation of cholesterol-lowering drugs until after completing endocrine therapy will yield a meaningful survival benefit is still an open-ended question. These data suggest that cholesterol-lowering statins could be prescribed as maintenance therapy after completing adjuvant endocrine therapy, since it has been established that statins also decrease 27HC concentrations [39]. The adverse impact of high CYP27A1 expression on survival was however non-significant among postmenopausal patients with ER+ tumors only in the BC-blood cohort, despite the significant association with poor survival in the overall population. Some possible explanations for the null findings could be the relatively shorter follow-up; median follow-up of only 7.0 years in BC-blood relative to 10.8 years in MDCS, which may not have been sufficiently long to investigate late events. The more generous use of adjuvant systemic therapies in the BC-blood resulted in fewer events to power the statistical analyses. Our previous study [39] evaluating the relationship between *CYP27A1* mRNA expression and prognosis in BC did not also find a significant association between *CYP27A1* with survival among patients > 50 years, and similar to the BC-blood cohort, the median follow-up was only 7.2 years in that study [39]. In addition, the age cut-off of 50 years used in the previous study [39] may not be an optimal proxy for menopausal status. More investigations are therefore required to confirm this time-dependent association between CYP27A1 expression and prognosis preferably in cohorts with reliable information regarding menopausal status and with extended follow-up.

The significantly higher RFS event rates associated with high intratumoral CYP27A1 expression in patients < 55 years (pre- and perimenopausal) presenting with ER+ tumors during the first 5 years after BC diagnosis in BC-blood is intriguing since the logical expectation is that high 27HC should display antagonistic properties under estrogenic conditions and therefore be protective in premenopausal patients, a premise which was verified in our previous study [39] reporting a significant improved RFS and OS for younger patients (< 50 years old) presenting with high compared with low *CYP27A1* mRNA in their tumors. Several studies have reported the benefit of suppressing ovarian function in premenopausal patients receiving endocrine therapies [48–50], and it is also well established that chemotherapy treatment can induce a postmenopausal physiological state in some premenopausal patients [51, 52]. The more generous prescription of chemotherapy in BC-blood and the higher likelihood that ovarian function suppressants may have been prescribed for patients in BC-blood given the more recent recruitment of patients into this cohort may to some extent explain the observed inverse association between high CYP27A1 and survival among the younger patients in this cohort. Additional differences in patients, tumor characteristics, and molecular assays used for measuring the expression of CYP27A1 between the studies (protein vs mRNA) may also have contributed to these divergent results. Unfortunately, with only 10% (*N* = 63) of the patients in MDCS under 55 years, it was impossible to perform any meaningful statistical evaluation among the younger patients, although a statistically non-significant trend showing a better survival for high CYP27A1 tumors was observed (data not shown). Though interesting, the limited number of cases and events included in this sub-group analysis in BC-blood warrants caution when interpreting these results. To better address this conundrum, an independent evaluation of CYP27A1 expression, both at the transcript and protein levels in the same patients in larger cohorts, is necessary to clarify the association between CYP27A1, menopausal status, and BC prognosis.

Although this observational study is based on two large population-based BC cohorts with clinical and tumor pathological data available for many conventional prognostic markers in BC, the results as presented may have been impacted by other important factors not mentioned above. Incorporation of other obesity-related factors besides body size measurements, lipid profiles in the tumor microenvironment and in the circulation, and complete treatment information into multivariable models in cohorts with extensive follow-up data will improve our appreciation of factors that influence endocrine therapy efficacy and the role of cholesterol metabolism in late recurrent disease pathobiology in postmenopausal patients.

## Conclusion

Except for serum cholesterol measurements, only a few clinical studies have directly addressed the prognostic role of genes associated with cholesterol metabolism in BC [12, 18, 30, 39]. A simple measurement of the serum cholesterol level cannot explain the molecular mechanism(s) driving tumor progression. Dysregulation of genes regulating cholesterol metabolism in the tumor might add value for prognostication and treatment prediction [15, 18]. Here, we present a novel biomarker of late lethal disease in postmenopausal ER+ BC, which is directly involved in cholesterol metabolism. Of relevance, these results provides indirect evidence that explains the associations between high cholesterol, obesity, and postmenopausal BC onset and progression seen in epidemiological studies and supports testing pharmacological drugs that decrease cholesterol metabolism and block CYP27A1 activity in postmenopausal patients with ER+ BC.

## Supplementary information

**Additional file 1:.** Supplementary table 1: Baseline patient and tumor characteristics in evaluable and non-evaluable patients.

**Additional file 2:.** Supplementary table 2: Associations between CYP27A1 expression and anthropometric factors in the BC-Blood cohort.

**Additional file 3:.** Supplementary table 3: Adjusted hazard ratios (95%CI) for overall survival and breast cancer-specific survival by tumor CYP27A1 expression in MDCS cohort over the full follow up period.

**Additional file 4:.** Supplementary table 4: Adjusted hazard ratios (95%CI) for overall survival and recurrence-free survival by tumor CYP27A1 expression in BC-blood cohort over the full follow up period.

## Data Availability

The data that support the findings from the Malmo Diet and Cancer cohort of this study are available on reasonable request from the corresponding authors (SK) and Signe Borgquist (SB).

## Abbreviations

27HC: 27-Hydroxycholesterol
ER+: Estrogen receptor-positive
BC: Breast cancer
CYP27A1: Cytochrome P450 family 27 subfamily A member 1
HR: Hazard ratio
CI: Confidence interval
OS: Overall survival
BCSS: Breast cancer-specific survival
RFS: Recurrence-free survival
MDCS: Malmö Diet and Cancer Study
BC-blood: Breast Cancer blood study
IHC: Immunohistochemistry
FFPE: Formalin-fixed paraffin-embedded
TMA: Tissue microarrays
OR: Odds ratio
IQR: Interquartile range
PgR: Progesterone receptor
AI: Aromatase inhibitor
